# Quality of life in adults with congenital adrenal hyperplasia relates to glucocorticoid treatment, adiposity and insulin resistance: United Kingdom Congenital adrenal Hyperplasia Adult Study Executive (CaHASE)

**DOI:** 10.1530/EJE-13-0128

**Published:** 2013-06

**Authors:** Thang S Han, Nils Krone, Debbie S Willis, Gerard S Conway, Stefanie Hahner, D Aled Rees, Roland H Stimson, Brian R Walker, Wiebke Arlt, Richard J Ross, the United Kingdom Congenital adrenal Hyperplasia Adult Study Executive (CaHASE)

**Affiliations:** 1 Department of Endocrinology, University College London Hospitals, London, UK; 2 School of Clinical and Experimental Medicine, Centre for Endocrinology, Diabetes and Metabolism, University of Birmingham, Birmingham, UK; 3 Society for Endocrinology, Bristol, UK; 4 Endocrinology and Diabetes Unit, Department of Medicine I, University of Würzburg, Würzburg, Germany; 5 Endocrinology Unit, Queen's Medical Research Institute, Centre for Cardiovascular Science, University of Edinburgh, Edinburgh, UK; 6 Institute for Molecular and Experimental Medicine, Centre for Endocrine and Diabetes Sciences, Cardiff University, Cardiff, UK; 7 Academic Unit of Diabetes, Endocrinology and Metabolism, University of Sheffield, Room 112, Floor M, Royal Hallamshire Hospital, Glossop Road, Sheffield, S10 2JF, UK

## Abstract

**Context:**

Quality of life (QoL) has been variously reported as normal or impaired in adults with congenital adrenal hyperplasia (CAH). To explore the reasons for this discrepancy we investigated the relationship between QoL, glucocorticoid treatment and other health outcomes in CAH adults.

**Methods:**

Cross-sectional analysis of 151 adults with 21-hydroxylase deficiency aged 18–69 years in whom QoL (assessed using the Short Form Health Survey), glucocorticoid regimen, anthropometric and metabolic measures were recorded. Relationships were examined between QoL, type of glucocorticoid (hydrocortisone, prednisolone and dexamethasone) and dose of glucocorticoid expressed as prednisolone dose equivalent (PreDEq). QoL was expressed as *z*-scores calculated from matched controls (14 430 subjects from UK population). Principal components analysis (PCA) was undertaken to identify clusters of associated clinical and biochemical features and the principal component (PC) scores used in regression analysis as predictor of QoL.

**Results:**

QoL scores were associated with type of glucocorticoid treatment for vitality (*P*=0.002) and mental health (*P*=0.011), with higher *z*-scores indicating better QoL in patients on hydrocortisone monotherapy (*P*<0.05). QoL did not relate to PreDEq or mutation severity. PCA identified three PCs (PC1, disease control; PC2, adiposity and insulin resistance and PC3, blood pressure and mutations) that explained 61% of the variance in observed variables. Stepwise multiple regression analysis demonstrated that PC2, reflecting adiposity and insulin resistance (waist circumference, serum triglycerides, homeostasis model assessment of insulin resistance and HDL-cholesterol), related to QoL scores, specifically impaired physical functioning, bodily pain, general health, Physical Component Summary Score (*P*<0.001) and vitality (*P*=0.002).

**Conclusions:**

Increased adiposity, insulin resistance and use of prednisolone or dexamethasone are associated with impaired QoL in adults with CAH. Intervention trials are required to establish whether choice of glucocorticoid treatment and/or weight loss can improve QoL in CAH adults.

## Introduction

Health-related quality of life (QoL) in adult patients with congenital adrenal hyperplasia (CAH) has been variously reported. Three groups have reported better, similar or mildly impaired QoL in CAH patients compared with controls or the normal population (1, 2, 3), while another three publications have reported poor QoL in CAH adults (4, 5, 6). The reason for this heterogeneity in QoL reporting for CAH adults is unclear but could relate to variables including treatment regimen, health care provision in different countries and genetics. We have recently reported poor QoL in a large cohort of UK adult patients with CAH (7). In this cohort we found that increasing glucocorticoid dose was associated with poor disease control and that dexamethasone treatment was associated with increased insulin resistance (8), but health outcomes were not associated with genotype (9). Overall, despite a large variety of glucocorticoid treatment regimens, androgen levels were generally poorly controlled and this was associated with an adverse metabolic profile including obesity. Similar observations of poor disease control, obesity and poor metabolic outcome have recently been reported in a large cohort of American CAH patients of whom 74 were adults (10). It is well established that obesity in the general adult population is related to a host of morbidities including impaired QoL (11, 12). Here, we have studied the relationship between QoL, glucocorticoid treatment regimen, disease control, and health measures including adiposity to better define the factors that associate with QoL in CAH adults.

## Materials and methods

### Patients

The UK Congenital adrenal Hyperplasia Adult Study Executive (CaHASE) cohort is a cross-sectional study of adult CAH patients, 18 years or older, recruited from 17 specialised British endocrinology centres. The study protocol was approved by West Midlands MREC (MREC/03/7/086) and registered with ClinicalTrials.gov (NCT00749593) (7). All participants gave written informed consent. One hundred and fifty-one subjects, all of whom were 21-hydroxylase deficient (50 males, 47 with classic and three with non-classic CAH; 101 females, 75 with classic and 26 with non-classic CAH), completed QoL questionnaires.

### Treatment

Treatment with hydrocortisone (*n*=41, 21 males and 20 females), prednisolone (*n*=65, 17 males and 48 females), hydrocortisone and prednisolone combination (*n*=8, one male and seven females) and any treatment regimen with dexamethasone (*n*=34, 11 males and 23 females) were included. The median duration of specific treatments was 10 years for hydrocortisone, 6.0 years for prednisolone, 1.0 year for hydrocortisone and prednisolone combination and 2.2 years for any treatment regimen with dexamethasone. For the purpose of some analyses treatment dose was converted to prednisolone dose equivalent (PreDEq) using the British National Formulary formula for comparing potency of glucocorticoids (13). This gives a ratio of prednisolone:hydrocortisone:dexamethasone of 1:4:0.15. PreDEq were grouped into the following categories for analysis: <5, 5–7.4, ≥7.5 mg.

### Quality of life

QoL was assessed by Short Form Health Survey (SF-36) using self-administered questionnaires. The SF-36 comprises health concepts measuring functioning (the ability to perform daily tasks and activities) and well-being (subjective internal states) of the subjects, including how people feel physically and emotionally and how they think and feel about their health. There are eight domains: physical functioning, role functioning limitations due to poor physical health, bodily pain, general health, vitality, social functioning, role functioning limitations due to poor emotional health and mental health. The first four domains were also grouped into Physical and the latter four domains grouped into Mental Component Summary Scores, derived from principal components analysis (PCA) of the SF-36 as previously described (14). Reference data for SF-36 scores were obtained from Prof. John Brazier (University of Sheffield, UK) comprising a representative random sample of 14 430 subjects from the UK population aged 18–79 years. For every CAH patient, 20 sex- and age-matched controls were randomly selected from the respective reference samples and *z*-scores generated. Adjustment for age and sex was performed by transformation of all domain score values from patients into age- (decade) and sex-adjusted *z*-scores.

### Health outcomes

Health outcomes included systolic blood pressure (SBP) and diastolic blood pressure (DBP), height, weight, waist circumference, biochemical assessment for fasting plasma glucose, serum insulin, total and HDL cholesterol and triglycerides, and steroid hormones including 17-hydroxyprogesterone (17-OHP), androstenedione and testosterone. All laboratories participate in the UK NEQAS scheme for quality control of biochemical assays. Patients were categorised into established mutation groups according to their genotype with the less severe mutation determining the group (9). Insulin resistance was calculated from the homeostasis model assessment of insulin resistance (HOMA-IR): insulin resistance=(fasting insulin (units/ml)×fasting glucose (mmol/l)/22.5).

### Statistical analysis

We looked at the relationship between QoL and other parameters using two different statistical methods: ANOVA with *post-hoc* analysis by least significant difference and PCA. ANOVA was used to investigate differences between groups, for example, those using different glucocorticoid treatment regimens. PCA was employed to reduce data by extracting only the important combinations of interrelated variables (principal components (PCs)) that account for a maximal amount of variance for a range of observed variables without losing much information from the larger set of original variables (15). The PCs are ordered so that the first one (PC1) captures the largest possible amount of variation in the original data. The respective component scores derived from the PCs may be used for further analysis as predictor variables in regression analysis against dependent variables such as used in our analysis: domains of QoL. PCA using 1.0 s as prior communality estimates (a communality refers to sum of squared loadings across variables) and the principal axis method was used to extract the components followed by a varimax (orthogonal) rotation. Components were retained for rotation if they fulfilled the Kaiser criterion (eigenvalues >1; indicating that >10% of the variance (*r*^2^) is extracted by a given component) (16) and scree test (17). In rotated component pattern, a variable was selected for a given PC if the factor loading was ≥0.50 for that PC, and <0.50 for the other. At least three variables that displayed a common construct were required to load on each PC. In this study PCA was carried out for ten variables of metabolic and disease control measures and mutation severity to derive three PCs (PC1, PC2 and PC3). The three PCs were used as predictor variables in stepwise multiple regression analysis against the dependent variables: age- and sex-adjusted *z*-scores for each of the eight domains of QoL and each of the Component Summary Scores. Results were presented as *F* statistic with 3 and 148 degrees of freedom between and within samples respectively for ANOVA and regression coefficients (*β*) and 95% CI for regression analysis. Analyses were performed using SPSS version 19.0 (SPSS, Inc.). Statistical significance was assumed when *P*<0.05.

## Results

**Figure 1:**
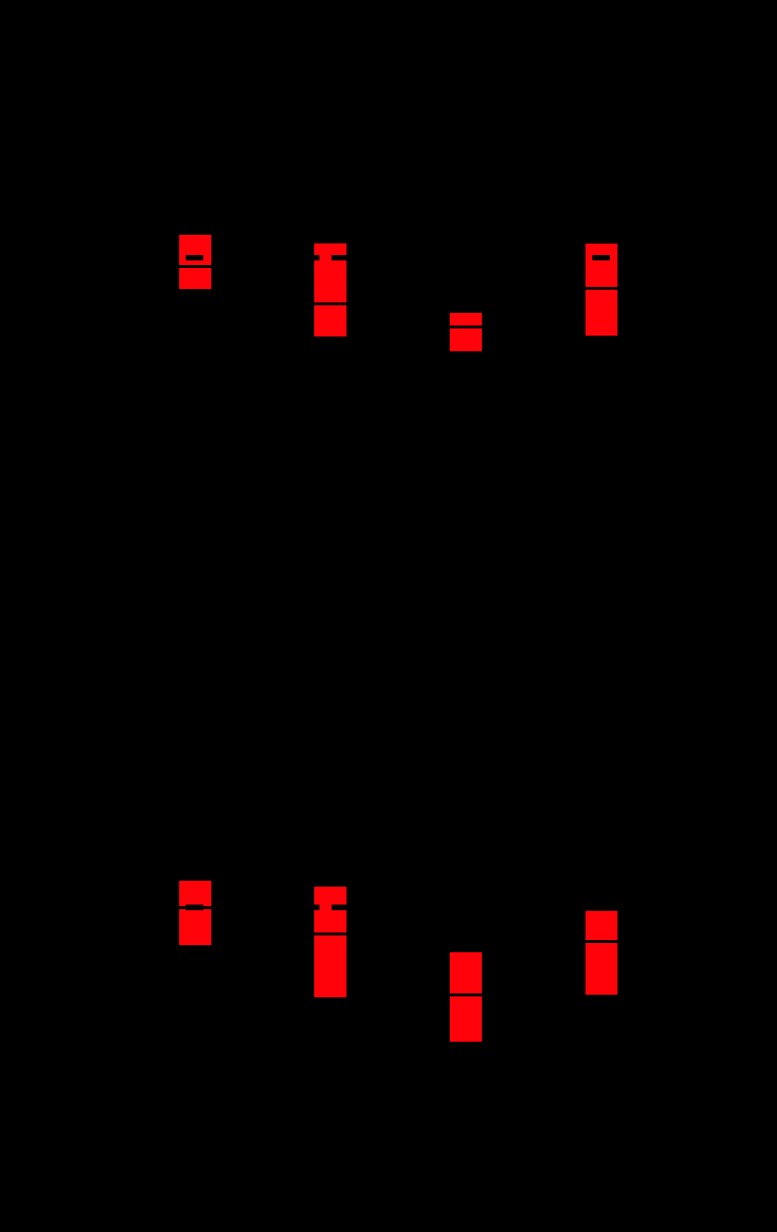
Association between type of glucocorticoid and QoL. Boxplots representing median and interquartile ranges of QoL (SF-36) *z*-scores of the two QoL domains, vitality (A) and mental health (B), for CAH patients using different type of glucocorticoid treatment; whiskers represent the 5th and 95th percentiles. A *z*-score of 0 represents the median of the reference population. *Post-hoc* analysis: compared with hydrocortisone-treated patients, *z*-scores for vitality and mental health were lower in those treated with a combination of hydrocortisone plus prednisolone (**P<0.01), any dexamethasone combination (*P<0.05) or prednisolone (**P<0.01, for vitality only). HC, hydrocortisone; Pred, prednisolone; Dex, dexamethasone.

Standardised scores and *z*-scores for the eight domains of QoL are presented in Table 1. Analysis of these domains between patients taking different types of glucocorticoid demonstrated a difference for vitality (ANOVA: *F*=5.1, *P*=0.002) and mental health (ANOVA: *F*=3.9, *P*=0.011). *Post-hoc* analysis revealed that compared with hydrocortisone monotherapy patients, vitality and mental health *z*-scores were lower in those taking combined hydrocortisone plus prednisolone therapy (*P*<0.01), prednisolone (*P*<0.01, for vitality only), and any regimen with dexamethasone (*P*<0.05) (Fig. 1). QoL did not correlate with PreDEq categories, mutation severity or classic vs non-classic phenotypes.

We then carried out a detailed PCA and extracted three PCs (PC1, PC2 and PC3) with observed eigenvalues of >1 that explained 61% of the total variance in the observed variables, with Kaiser-Meyer-Olkin measure of sampling adequacy (0.66) and Bartlett's test of sphericity (*χ*^2^=170, *P*<0.001). PC1 reflects ‘disease control’ comprising androstenedione, 17-OHP and testosterone. PC2 reflects ‘adiposity and insulin resistance’ comprising waist circumference, serum triglycerides, HOMA-IR and HDL-cholesterol. PC3 reflects ‘blood pressure and mutations’ comprising SBP, DBP and mutations.

**Figure 2:**
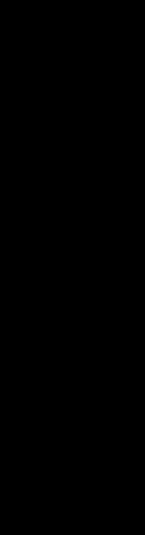
Association between individual components of the adiposity and insulin resistance (PC2) and physical functioning QoL. Error plots with mean and 95% confidence limits showing the size of waist circumference (A), levels of triglycerides (B), HDL cholesterol (C) and HOMA-IR (D) according to the physical functioning domain categorised into three groups based on *z*-scores ≥0 (*n*=69), <0 to −1 (*n*=36) and <−1 (*n*=46). *Post-hoc* analysis: **P<0.01, ***P<0.001 compared with group with *z*-score ≥0.

**Figure 3:**
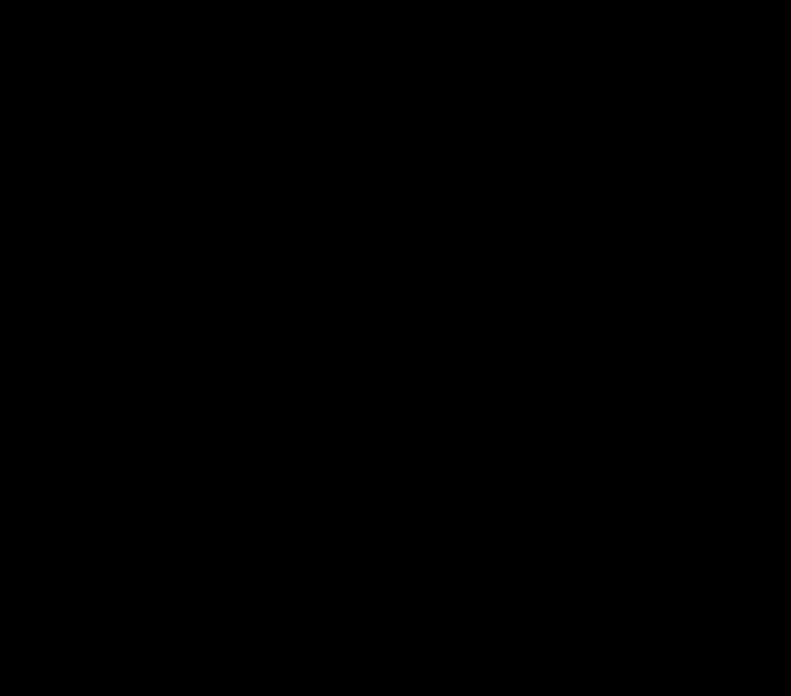
Association between individual components of the adiposity and insulin resistance (PC2) and vitality QoL. Error plots with mean and 95% confidence limits showing the size of waist circumference (A), levels of triglycerides (B), HDL cholesterol (C) and HOMA-IR (D) according to the vitality domain categorised into three groups based on *z*-scores ≥0 (*n*=69), <0 to −1 (*n*=36) and <−1 (*n*=46). *Post-hoc* analysis: *P<0.05, **P<0.01 compared with group with *z*-score ≥0.

The resulting PC scores obtained from PCA were analyzed by stepwise multiple regression analysis (adjusted for PreDEq and glucocorticoid regimens) to assess their relationship with each of the eight domains of QoL *z*-scores and each of the two Component Summary Scores. PC1 ‘disease control’ and PC3 ‘blood pressure and mutations’ did not associate with any of the QoL domains. By contrast, PC2, reflecting ‘obesity and insulin resistance’, related to impaired physical functioning (*β*=−0.72, 95% CI: −1.11 to −0.35, *r*^2^=19.9%, *P*<0.001), bodily pain (*β*=−0.55, 95% CI: −0.82 to −0.28, *r*^2^=21.6%, *P*<0.001), general health (*β*=−0.50, 95% CI: −0.80 to −0.20, *r*^2^=16.0%, *P*=0.001), vitality (*β*=−0.40, 95% CI: −0.65 to −0.16, *r*^2^=15.5%, *P*=0.002) and Physical Component Summary Score (*β*=−0.58, 95% CI: −0.83 to −0.33, *r*^2^=26.4%, *P*<0.001) (Table 2). This relationship has not influenced PreDEq, type of glucocorticoid replacement (hydrocortisone, prednisolone and dexamethasone) and their respective doses and treatment regimens (dosage frequency: once daily, twice daily, thrice daily and timing of administration: circadian patterns). Figures 2 and 3 show the components of PC2, reflecting ‘obesity and insulin resistance’, plotted against the scores for physical functioning and vitality that illustrates the observed poorer health outcome measures in patients with lower QoL *z*-scores.

## Discussion

We found QoL to be worse in adult CAH patients using prednisolone and dexamethasone compared with those only taking hydrocortisone. We also found that markers of adiposity and insulin resistance (waist circumference, serum triglycerides, HOMA-IR and HDL-cholesterol) were associated with a worse QoL. Combining these QoL data with our previous analysis of metabolic parameters (8) in this cohort of UK patients suggests that treatment with more potent glucocorticoids or combination therapy is associated with a poor metabolic profile and impaired QoL. It is not possible to infer from our results what is cause or effect. Patients who have poor disease control and impaired QoL may be those that are selected for more potent glucocorticoids, or the treatment itself may reduce QoL.

Our study is the largest published cohort of CAH adults and the first to report on the relationship between QoL, glucocorticoid treatment and health outcomes. Our QoL results are at variance with some previous reports. In a nationwide Finnish study, 108 CAH patients were identified; these, 58 were over 16 years, and 32 completed a QoL questionnaire (RAND-36) (1). In this subgroup of patients QoL was significantly better than the reference Finnish population and commentators speculated this was because of greater physician–patient contact. In a study of 81 German CAH patients from two tertiary care centres the patients had impaired health-related QoL in three of five GBB-24 scores whereas SF-36 and Hospital Anxiety and Depression Scores (HADS) did not differ from controls, and QoL was significantly better than a comparator group of patients with primary adrenal insufficiency (2). As both adrenal insufficiency and CAH patient groups receive glucocorticoids the authors suggested that factors other than hormone replacement therapy could account for the difference in QoL. One factor they discuss is that patients with a congenital disease may have a different perception of QoL to those with an acquired condition. In another cohort of German patients, from a single clinic, QoL (EDLQ) in 45 adult female CAH patients was similar to control subjects who were staff at the same hospital (3). The authors considered that differences found regarding social support and illness processing may be mechanisms patients have developed to help them to cope with their condition. In contrast to these three reports, our results showed that QoL of CAH adults is impaired in comparison with QoL in the general population and that impaired QoL is related to type of glucocorticoid treatment. One potential reason for the difference in results is that our cohort of patients was managed in 17 different centres, using a variety of treatment regimens and with a great spectrum of disease control. Some patients appeared over-treated, based on suppressed androgens, and some patients under-treated, based on raised androgens. In contrast, the previously reported studies either come from a selected subgroup of patients or from single centres where patients were treated by a relatively small number of clinicians, who may have obtained tighter control of their disease. However, our findings of impaired QoL in adults with CAH are consistent with some other studies. In a cross-sectional study of 104 Norwegian patients, QoL was impaired in all the SF-36 subdomains and most pronounced for general health and vitality perception (5). In this cohort, working disability was reported by 19% of the patients, compared with 10% in the general population.

We found that QoL in adults with CAH related to adiposity and insulin resistance. This has not been reported previously, but is recognised in the general population where QoL, as measured by SF-36, is impaired in people with increased adiposity (11, 12) and insulin resistance (18). We observed that increased adiposity, and its consequent insulin resistance, relates to the three ‘physical’ QoL domains of the SF-36 (physical functioning, bodily pain and general health) but only to the vitality domain of the ‘mental health’ criteria. This distinction was further confirmed in our analysis where adiposity and insulin resistance were inversely related to the Physical Component Summary Score but not the Mental Component Summary Score of SF-36. The results are consistent with the observation that increased adiposity and insulin resistance in European populations relate primarily to adverse physical health rather than mental health criteria of QoL (11, 12, 18). In PCA analysis of SF-36, although the vitality domain was retained by the Mental Component Summary Score, its loading (correlation, *r*=0.654) on this component is not as strong as the other three domains (social functioning, role functioning limitations due to poor emotional health and mental health: *r*≥0.825). In fact, the vitality domain also loads on the Physical Component Summary Score but with less weight (correlation, *r*=0.515). This finding is similar to previous published results obtained from a general USA population sample showing vitality to correlate with both Mental (0.64) and Physical Component Summary Scores (0.47) (14). The two Component Summary Scores therefore are not exclusively independent and should be interpreted with the individual QoL domains as we have done in our analysis (14, 19).

QoL did not relate to genotype (mutations) (9) or phenotype (classic or non-classic) (7). This observation supports the concept that the impaired QoL is acquired and may in part relate to type of glucocorticoid treatment (i.e. adverse effects from prednisolone or dexamethasone) or reflect the patients' poor disease control.

Limitations in this study include its cross-sectional nature and the large number of variables examined. It is difficult to quantify the absolute exposure and duration of specific glucocorticoid regimens. It is likely that most patients were treated with hydrocortisone during childhood (20). The duration of glucocorticoid treatment was recorded with the median for each type of treatment ranging between 1 and 10 years. To address the large number of variables within this database, PCA was employed as it allowed us to examine associations in large and complex multivariate databases that requires no a priori assumptions about the data and is thus ideal for exploratory data analysis (15). Future prospective studies designed specifically to examine the long-term effects of glucocorticoid therapy in CAH patients are needed to address the limitations of this study.

In conclusion, increased adiposity, insulin resistance and the use of combination glucocorticoid therapy with prednisolone and dexamethasone are associated with impaired QoL in adults with CAH independently of mutation severity. However, whether obesity or treatment results in poor QoL or *vice versa* is yet to be established. Further studies, ideally randomised controlled intervention trials, are required to establish whether choice of glucocorticoid treatment and/or weight loss can improve QoL in CAH adults.

## Figures and Tables

**Table 1:** QoL SF-36 standardised scores and *z*-scores for CAH adults, based on reference data obtained from Prof. John Brazier (University of Sheffield, UK) comprising a representative random sample of 14 430 subjects from the UK population aged 18–79 years. For every CAH patient, 20 sex- and age-matched controls were randomly selected from the respective reference samples and *z*-scores generated. Adjustment for age and sex was done by transforming all domain score values from patients into age- (decade) and sex-adjusted *z*-scores.

	**Standardised scores** (mean±s.d.)	***Z*-scores** (mean±s.d.)
Physical function	81.2±25.2	−0.74±1.53
Role limitations due to physical problems	74.2±37.7	−0.71±1.33
Bodily pain	68.4±27.8	−0.45±1.26
General health	56.1±25.4	−0.61±1.09
Vitality	50.4±21.6	−0.87±1.21
Social functioning	73.2±26.9	−0.54±1.02
Role limitations due to emotional problems	69.3±40.1	−0.91±1.66
Mental health	65.9±20.9	−0.72±1.19

**Table 2:** Regression coefficients (*β*) and explained variances (*r*^2^) obtained from stepwise multiple regression analysis using the three principal components (PC1, PC2 and PC3) as predictor variables and health-related QoL SF-36 questionnaire age- and sex-adjusted *z*-scores as dependent variables. Only PC2, reflecting adiposity and insulin resistance (waist circumference, serum triglycerides, HOMA-IR and HDL-cholesterol), was retained showing adverse relationships with physical function, bodily pain, general health, vitality and Physical Component Summary Score. Additional adjustment for PreDEq or type of glucocorticoid replacement (hydrocortisone, prednisolone and dexamethasone) did not change these relationships.

	**Predictor variable** (PC2, adiposity and insulin resistance)		
**Dependent variables**	*β* (95% CI)	*P*	*r*^2^ (%)
Physical function	−0.72 (−1.11 to −0.35)	<0.001	19.9
Role limitations due to physical problems	NS	–	–
Bodily pain	−0.55 (−0.82 to −0.28)	<0.001	21.6
General health	−0.50 (−0.80 to −0.20)	0.001	16.0
Physical Component Summary Score^a^	−0.58 (−0.83 to −0.33)	<0.001	26.4
Vitality	−0.40 (−0.65 to −0.16)	0.002	15.5
Social functioning	NS	–	–
Role limitations due to emotional problems	NS	–	–
Mental health	NS	–	–
Mental Component Summary Score^b^	NS	–	–

NS, Not significant.

a Physical Component Summary Score comprises four of the eight QoL domains: physical functioning, role functioning limitations due to poor physical problems, bodily pain and general health.

b Mental Component Summary Score comprises the other four QoL domains: vitality, social functioning, role functioning limitations due to poor emotional health and mental health.
